# Estimation of the mechanical properties of the eye through the study of its vibrational modes

**DOI:** 10.1371/journal.pone.0183892

**Published:** 2017-09-18

**Authors:** M. Á. Aloy, J. E. Adsuara, P. Cerdá-Durán, M. Obergaulinger, J. J. Esteve-Taboada, T. Ferrer-Blasco, R. Montés-Micó

**Affiliations:** 1 Department of Astronomy and Astrophysics, University of Valencia, 46100 Burjassot, Spain; 2 Department of Optics and Optometry and Vision Sciences, University of Valencia, 46100 Burjassot, Spain; Universidade do Minho, PORTUGAL

## Abstract

Measuring the eye’s mechanical properties in vivo and with minimally invasive techniques can be the key for individualized solutions to a number of eye pathologies. The development of such techniques largely relies on a computational modelling of the eyeball and, it optimally requires the synergic interplay between experimentation and numerical simulation. In Astrophysics and Geophysics the remote measurement of structural properties of the systems of their realm is performed on the basis of (helio-)seismic techniques. As a biomechanical system, the eyeball possesses normal vibrational modes encompassing rich information about its structure and mechanical properties. However, the integral analysis of the eyeball vibrational modes has not been performed yet. Here we develop a new finite difference method to compute both the spheroidal and, specially, the toroidal eigenfrequencies of the human eye. Using this numerical model, we show that the vibrational eigenfrequencies of the human eye fall in the interval 100 Hz–10 MHz. We find that compressible vibrational modes may release a trace on high frequency changes of the intraocular pressure, while incompressible normal modes could be registered analyzing the scattering pattern that the motions of the vitreous humour leave on the retina. Existing contact lenses with embebed devices operating at high sampling frequency could be used to register the microfluctuations of the eyeball shape we obtain. We advance that an inverse problem to obtain the mechanical properties of a given eye (e.g., Young’s modulus, Poisson ratio) measuring its normal frequencies is doable. These measurements can be done using non-invasive techniques, opening very interesting perspectives to estimate the mechanical properties of eyes *in vivo*. Future research might relate various ocular pathologies with anomalies in measured vibrational frequencies of the eye.

## Introduction

Obtaining the mechanical properties of the human eye is fundamental for the future development of artificial materials that can be employed as substitutes for natural tissues [1]. Measuring the eye’s mechanical properties *in vivo* and with minimally invasive techniques can be the key for individualized solutions to a number of eye pathologies. The development of such techniques largely relies on a computational modelling of the eyeball [2] and, it optimally requires the synergic interplay between experimentation and numerical simulation [3].

The eye is a complex organ consisting of several functional and mutually interacting parts [4]. The most important ones from the mechanical point of view are the cornea, lens, vitreous, sclera and retina. Each of these elements holds distinctive mechanical properties that are closely related to their respective anatomic functionality. Changes in the mechanical properties may entail a number of pathologies or even a loss of functionality [5]. Reciprocally, damage inflicted to a healthy eye may result in changes in its elastic and mechanical properties [6]. The mechanical modelling of the human eye is a field that has gained relevance to rationalize the physiology and pathology of the eye [3]. The field is exponentially developing pace to pace with our ability of implementing more complex models on modern computers [7]. Our knowledge of the mechanical properties of the eye has basically come through three different ways: experimentation, *in vivo* monitoring, or computational modelling. We develop our work in the later framework.

Measuring the elasticity properties of the different tissues forming an eye is challenging. Very often, the determination of mechanical properties of the eye results from a mechanical interaction with its different parts [8–10]. In addition to standard mechanical testing, the cornea has been characterized through high-resolution microscopy techniques [11], as well as with the Ocular Response Analyzer [12, 13]. Likewise, multiple studies have examined the overall biomechanical properties of the sclera [14, 15]. Ultrasound biomicroscopy has been used to measure the scleral thickness [16, 17]. Magnetic Resonance Imaging (MRI) techniques applied *in vivo* resulted inaccurate because of the random eye movement of the patients, though it is possible to use MRI scans to produce 3D models of the corneoscleral shells in post-mortem patients [18].

Novel non-invasive techniques need to be devised to measure the mechanical properties of the human eye. Here we show that these properties are related to the normal vibrational modes of the eyeball, i.e., to the periodic variations of matter inside of the eyeball resulting from perturbations with respect to its equilibrium state. A germane idea, but restricted to the corneal biomechanics, has been addressed in other works considering the vibrational properties of the cornea. Employing the dispersive properties of Lamb waves, Zhang et al. [19] assess the viscoelastic properties of *ex-vivo* bobine corneas. The vibration analysis of the cornea has also been the subject of a handful of very recent publications [20–22]. Kling S. et al. [20], show simulations of deformation and vibration of the cornea focusing on the impact of a number of parameters as, e.g., different intraocular pressures and corneal elastic and viscoelastic properties. These authors built a sophisticated viscoelastic finite element model that predicts the experimental corneal deformation response to an air-puff for different conditions. Later, Kling et al. [21] analysed the corneal deformation vibrations in terms of the numerical model applied to data from optical coherence tomography. Their model predicted response vibrational corneal frequencies in the range 50–510 Hz.

Remarkably, the previous works have focused on the vibrational deformations of the eyeball shape (*spheroidal modes*), but not on periodic motions, which do not involve radial displacements of the inner constituents of the eye (*toroidal modes*), specially the vitreous humor. As we shall see, toroidal modes also encode valuable information about the biomechanics of the eyeball and smaller frequencies than the commonly studied spheroidal modes. We have been inspired by the extensive use of the remote measurements of normal-mode related physical quantities in Geophysics and Astrophysics. For instance, the solar interior is routinely scanned by means of helioseismic techniques, which are based on the measurement of the global resonant oscillations of the Sun [23]. Likewise, employing the principles of asteroseismology, neutron star interiors are proven [24, 25] in an attempt to decipher the equation of state for matter at nuclear densities. We believe that this treatment opens up a new set of techniques for remote measuring of the eyes structural properties.

## Materials and methods

The oscillations under consideration in our model are free elastic vibrations, which we assume may arise when applying generic stresses, e.g., on the sclera or the cornea. We tackle the numerical calculation of the vibrational eigenfrequencies and eigenmodes of the human eye under a number of simplifying assumptions. In an initial approximation to the problem, we model the eyeball as a *spherical*, *homogeneous* and *isotropic* *elastic* solid ball with axial symmetry. While assuming that the eyeball is axially symmetric is very well justified, the assumptions of homogeneity and isotropy are certainly not the most accurate possible. However, these assumptions serve for the primary purpose of reducing the dependence of the constitutive equation only to two elastic constants or moduli of the eye material: the Young’s modulus *E*, and the Poisson ratio *σ*. In this simplified framework, we will compute, first analytically and afterwards numerically, the eigenfrequencies of the model attempting to grasp the essential mechanics of an average human eye. In a second step, we model the eyeball differentiating the corneo-scleral layer from an assumed isotropic interior (model A below). Finally, a more elaborated eyeball model in axial symmetry is built and its normal toroidal modes computed (model B below).

## Results

**Fig 1:**
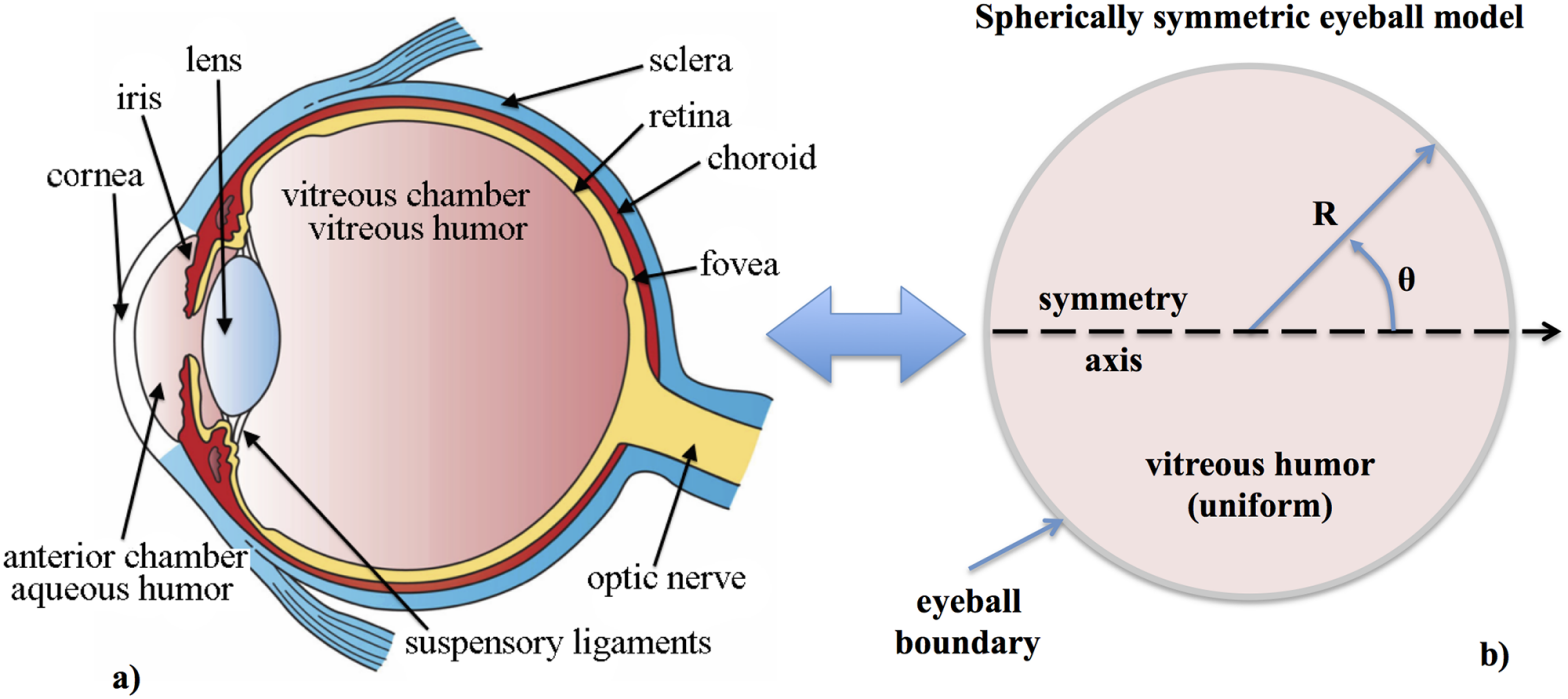
Simplified mechanical model of the eyeball. Left: transversal cut of the human eye with the different structural parts annotated in it (source: Wikipedia). Right: spherically symmetric, homogeneous and isotropic eyeball model employed in this work.

As we have mentioned above, we first model the eyeball as a spherically symmetric, homogeneous and isotropic elastic solid ball (Fig 1). This simplification allows us to use known analytical solutions (S1 Appendix) in other physics disciplines (e.g., seismology [26] or gravitational wave physics [27, 28]) to calibrate our numerical code (described in Sect. Numerical code).

### Numerical code

Since we aim to employ non-trivial boundary conditions, we are forced to solve numerically the eigenvalue problem at hand. We have developed a code that solves the eigenvalue problem set by the Navier-Cauchy equation discretizing the eyeball sphere on a two-dimensional grid of nodes in spherical coordinates (0 ≤ *r* ≤ *R*, 0 ≤ *θ* ≤ *π*). As a first step, we have assumed the elastic moduli to be uniform throughout the spatial grid. However, there is no restriction to implement elastic moduli that depend on the location in the eyeball. This is important because it enables us to improve the degree of realism of our model for the vibrational modes of the eye, in particular, by using different elastic moduli for the sclera, the cornea, the lens, and the vitreous humour.

The normal frequencies of the eyeball can be obtained as an eigenvalue problem (see Eq (6) of the Section Supporting information). In spherical coordinates, and under the assumption of axisymmetry, i.e., neglecting the *φ*-dependence, the displacements can be written as

$$\begin{array}{c} u_{i}=\left(u_{r}\left(r,\theta \right),u_{\theta }\left(r,\theta \right),u_{\varphi }\left(r,\theta \right)\right). \end{array}$$

Using the Einstein summation convention for repeated indices and the “_;*j*_” notation to express the covariant derivative with respect to the *j* coordinate (*j* = {*r*, *θ*, *ϕ*}), the stress tensor reads

$$\begin{array}{c} \sigma_{ij}=\mu \left(u_{i;j}+u_{j;i}\right)+\lambda u_{l;l}\delta_{ij}, \end{array}$$ (1)

which satisfies the equation

$$\begin{array}{c} \sigma_{ij;j}=\rho \;p^{2}u_{i}. \end{array}$$ (2)

In components this equation can be written as

$$\begin{array}{c} -\frac{\mu }{\rho }\Delta u_{r}-\frac{\lambda +\mu }{\rho }u_{j;jr}+\mu_{;j}\left(u_{r;j}+u_{j;r}\right)+\lambda_{;r}u_{l;l}=p^{2}u_{r} \end{array}$$ (3)

$$\begin{array}{c} -\frac{\mu }{\rho }\Delta u_{\theta }-\frac{\lambda +\mu }{\rho }u_{j;j\theta }+\mu_{;j}\left(u_{\theta ;j}+u_{j;\theta }\right)+\lambda_{;\theta }u_{l;l}=p^{2}u_{\theta } \end{array}$$ (4)

$$\begin{array}{c} -\frac{\mu }{\rho }\Delta u_{\varphi }-\frac{\lambda +\mu }{\rho }u_{j;j\varphi }+\mu_{;j}\left(u_{\varphi ;j}+u_{j;\varphi }\right)+\lambda_{;\varphi }u_{l;l}=p^{2}u_{\varphi }, \end{array}$$ (5)

where we have considered that the *Lamé constants* *μ* and λ (see S1 Appendix for the relation of these constants with *E* and *σ*) can vary spatially. Because of the assumed axial symmetry, all the derivatives with respect to *φ* vanish, and Eq (5) decouples from Eqs (3) and (4), yielding the following equation for the toroidal modes:

$$\begin{array}{c} -\frac{\mu }{\rho }\Delta u_{\varphi }+\partial_{r}\mu \partial_{r}u_{\varphi }+\frac{1}{r^{2}}\partial_{\theta }\mu \partial_{\theta }u_{\varphi }-(\frac{\partial_{r}\mu }{r}+\frac{\partial_{\theta }\mu }{r^{2}}\cot\theta )u_{\varphi }=p^{2}u_{\varphi }, \end{array}$$ (6)

if zero traction boundary conditions, *σ*_*rφ*_ = 0, are imposed. The spheroidal modes result from Eqs (3) and (4):

$$\begin{array}{ccc} -\frac{\mu }{\rho }\Delta u_{r}-\frac{\lambda +\mu }{\rho }\partial_{r}[\frac{1}{r^{2}}\partial_{r}r^{2}u_{r}+\frac{1}{r\sin\theta }\partial_{\theta }\sin\theta u_{\theta }] & & \\ +[2\partial_{r}\mu \partial_{r}u_{r}+\frac{1}{r}\partial_{\theta }\mu (\partial_{r}u_{\theta }+\frac{1}{r}\partial_{\theta }u_{r}-\frac{1}{r}u_{\theta })]+\partial_{r}\left(\nabla \cdot u\right) & \;=\; & p^{2}u_{r} \end{array}$$ (7)

$$\begin{array}{ccc} -\frac{\mu }{\rho }\Delta u_{\theta }-\frac{\lambda +\mu }{r\rho }\partial_{\theta }[\frac{1}{r^{2}}\partial_{r}r^{2}u_{r}+\frac{1}{r\sin\theta }\partial_{\theta }\sin\theta u_{\theta }] & & \\ +[\partial_{r}\mu (\partial_{r}u_{\theta }+\frac{1}{r}\partial_{\theta }u_{r}-\frac{1}{r}u_{\theta })+\frac{2}{r}\partial_{\theta }\mu (\frac{1}{r}\partial_{\theta }u_{\theta }+\frac{u_{r}}{r})]+\frac{1}{r}\partial_{\theta }\left(\nabla \cdot u\right) & \;=\; & p^{2}u_{\theta } \end{array}$$ (8)

also imposing zero traction boundary conditions, *σ*_*rr*_ = *σ*_*rθ*_ = 0 (see below) and making use of the replacement *u*_*l*;*l*_ = ∇ ⋅ *u*. Eqs (7) and (8) can be cast as an eigenvalue equation, $Lu=\tilde{\lambda }u$, for the following vectorial operator and $\tilde{\lambda }$ is a generic eigenvalue of *L*. In matrix form we have:

$$\begin{array}{c} Lu=[\begin{array}{cc} a_{rr} & a_{r\theta }\\ a_{\theta r} & a_{\theta \theta } \end{array}][\begin{array}{c} u_{r}\\ u_{\theta } \end{array}]\;, \end{array}$$ (9)

where

$$\begin{array}{ccc} a_{rr} & ≔ & -\frac{\lambda +2\mu }{\rho }\partial_{rr}-\frac{2(\lambda +2\mu )}{\rho r}\partial_{r}-\frac{\mu }{\rho r^{2}}\partial_{\theta \theta }-\frac{\mu \cot\theta }{\rho r^{2}}\partial_{\theta }+\frac{2(\lambda +2\mu )}{\rho r^{2}}\\ & & +\left(2\partial_{r}\mu +\partial_{r}\lambda \right)\partial_{r}+\frac{1}{r^{2}}\partial_{\theta }\mu \partial_{\theta }+\frac{2}{r}\partial_{r}\lambda \end{array}$$ (10)

$$\begin{array}{ccc} a_{r\theta } & ≔ & -\frac{(\lambda +\mu )\cot\theta }{\rho r}\partial_{r}+\frac{\lambda +3\mu }{\rho r^{2}}\partial_{\theta }-\frac{\lambda +\mu }{\rho r}\partial_{r\theta }+\frac{\cot\theta (\lambda +3\mu )}{\rho r^{2}}\\ & & +\frac{1}{r}\partial_{\theta }\mu \partial_{r}+\frac{1}{r}\partial_{r}\lambda \partial_{\theta }+(\frac{\cot\theta }{r}\partial_{r}\lambda -\frac{1}{r^{2}}\partial_{\theta }\mu ) \end{array}$$ (11)

$$\begin{array}{ccc} a_{\theta r} & ≔ & \frac{2(\lambda +2\mu )}{\rho r^{2}}\partial_{\theta }-\frac{\lambda +\mu }{\rho r}\partial_{r\theta }\\ & & +\frac{1}{r}\partial_{\theta }\lambda \partial_{r}+\frac{\partial_{r}\mu }{r}\partial_{\theta }+\frac{1}{r^{2}}(\partial_{\theta }\mu +2\partial_{\theta }\lambda ) \end{array}$$ (12)

$$\begin{array}{ccc} a_{\theta \theta } & ≔ & -\frac{\mu }{\rho }\partial_{rr}-\frac{2\mu }{\rho r}\partial_{r}+-\frac{\lambda +2\mu }{\rho r^{2}}\partial_{\theta \theta }-\frac{(\lambda +2\mu )\cot\theta }{\rho r^{2}}\partial_{\theta }+\frac{\left(\lambda +2\mu \right)\csc^{2}\;\theta }{\rho r^{2}}\\ & & +\partial_{r}\mu \partial_{r}+\frac{1}{r^{2}}\left(\partial_{\theta }\mu +\partial_{\theta }\lambda \right)\partial_{\theta }+(\frac{1}{r^{2}}\cot\theta \partial_{\theta }\lambda -\frac{1}{r}\partial_{r}\mu ) \end{array}$$ (13)

The explicit expression for the zero traction boundary conditions in spherical coordinates is

$$\begin{array}{ccc} \sigma_{rr} & = & \left(2\mu +\lambda \right)\partial_{r}u_{r}+\frac{2\lambda }{r}u_{r}+\frac{\lambda }{r}\left(\partial_{\theta }u_{\theta }+\cot\theta u_{\theta }\right)=0 \end{array}$$ (14)

$$\begin{array}{ccc} \sigma_{r\theta } & = & \mu r\partial_{r}(\frac{u_{\theta }}{r})+\frac{\mu }{r}\partial_{\theta }u_{r}=0 \end{array}$$ (15)

$$\begin{array}{ccc} \sigma_{r\varphi } & = & \mu r\partial (\frac{u_{\varphi }}{r})=0. \end{array}$$ (16)

In both types of modes, we compute in a first step the eigenvalues (vibrational frequencies), and in a second step the eigenfunctions (normal displacements). For the eigenvalues we simply compute the zeros of the characteristic polynomial. In practice, working in logarithmic space is advantageous because it reduces the magnitude of coefficients of the polynomial. Knowing the family of eigenvalues, we compute the kernel for each one of them. We substitute each eigenvalue into the corresponding equation, Eq (6) or Eqs (7) and (8), obtaining an elliptic equation. With this, we subtract the eigenvalue from the diagonal of the matrix produced by the discretization of the elliptic operator, and proceed further solving the corresponding system of equations by direct numerical inversion of the matrix of the system. As the rank of this matrix cannot be complete, we will obtain the compatible but indeterminate solution as a function of some of the variables (either one or two variables for the toroidal and the spheroidal case, respectively).

Finally, we outline the fact that our finite difference method to solve for the eigenfrequencies of the *L* operator is based on the standard LAPACK package. In future upgrades of our methodology we will make use of suitably adapted methods from [29, 30]. These newly developed iterative methods are computationally very efficient and have been even implemented on GPUs [31]. On a single core of a standard laptop it takes ≲ 5 minutes to compute the spectrum of eigenvalues of *L*. Due to the high-degree of parallelism of the method, a complete solution can be obtained in a matter of few seconds in devices harbouring a standard GPU. This efficiency can be employed for future devices requiring real-time solutions.

#### Code calibration

We calibrate the code by comparing the frequencies computed with our numerical code and the corresponding analytic values at a density of *ρ* = 1 kg m^−3^, an elastic moduli of *E* = 2.5 Pa, *σ* = 0.25 and a radius of the sphere of *R* = 1m. Note that these values do not correspond to a typical human eye. They are employed for numerical convenience.

**Fig 2:**
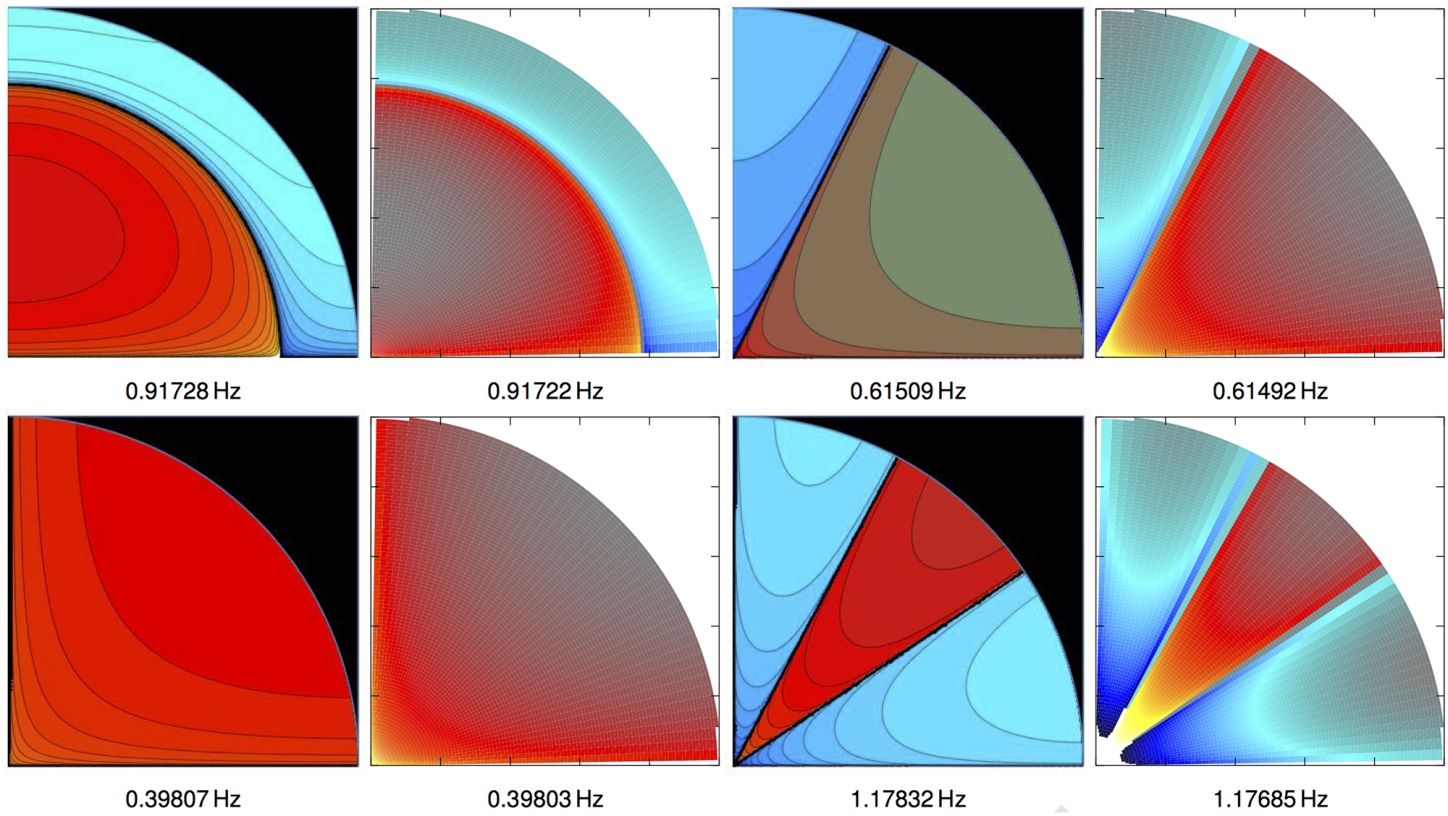
Calibration of the method. Comparison between the analytic (panels with black background) and numerical (white background) solutions of vibrational patterns. Because of the symmetries, only one quadrant of the full equatorial plane of an spherical body is shown. Modes of odd and even parities are displayed in the upper and lower panels, respectively. In this case, we are using 100 points in the radial direction and 50 in the angular one. We can also observe a good agreement in their corresponding frequencies (listed below each panel), that improves as we increase the resolution.

As shown in Fig 2, we get a good agreement in the toroidal (*φ*−) case, both in the vibrational patterns and in their corresponding frequencies, demonstrating the ability of the numerical code to recover the analytic values. We point out that agreement improves with a finer mesh encompassing the eyeball (in Fig 2 we employ a relatively coarse grid of 100 × 50 points in the *r* × *φ* directions for our finite-difference method). A similar analysis has been done for modes where the displacements of the material happen only in the *r*− and *θ*−directions (spheroidal modes). The conclusion of both calibration experiments is that our numerical procedure to compute the eigenfrequencies of the system and their displacements is accurate enough.

#### Application of the method to a (simplified) typical human eye

The exact eigenfrequency values are sensitive to the imposed boundary conditions. We assume that the surface of the eye (either the sclera or the cornea) is free to oscillate when suitable perturbations are inflicted to the eyeball. These perturbations can be originated by the muscles acting either on the outer eyeball surface or on the lens during the accommodation (e.g., contraction of the ciliary body due to stimulation of the autonomic nervous system). Here, we consider a set of “standard” eye parameters. We adopt *R* = 0.0125 m, *ρ* = 1000 kg m^−3^ for the eyeball typical radius and average density, respectively. Mean values for the corneal and scleral Poisson ratio, *σ*, are in the range 0.42–0.47 [2]. We take *σ* = 0.49, slightly above the average to account for the incompressible character of the vitreous humour. As the eigenfrequencies are roughly proportional to *σ*^−1/2^, their predicted values are basically insensitive to this parameter in the typical ranges measured for constituents of the human eye. There is a large scattering in the values of the Young’s modulus, *E*, of different parts of the eye [6]. We employ a typical value *E* = 0.2985 MPa. The eigenfrequencies exhibit a weak dependence with the value of the Young’s modulus, ∝ *E*^1/2^. Since the largest values reported for the Young’s modulus are *E*_max_ ≃ 20 MPa, at most a factor of a few increase in the computed frequencies is possible. Hereafter, we will refer to this simplified, spherically symmetric model as model S0.

**Fig 3:**
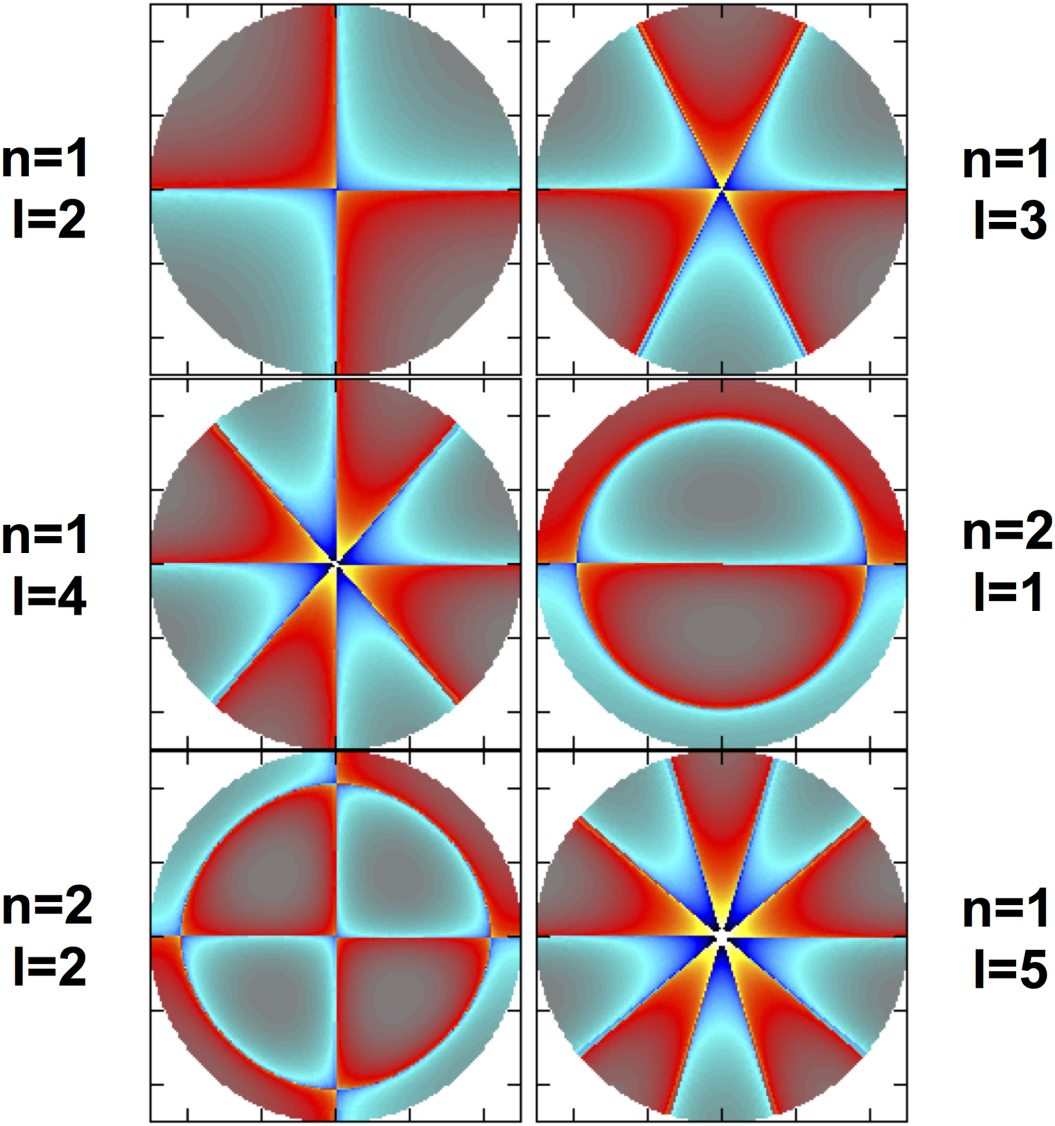
Toroidal vibrational modes. Six different patterns of toroidal vibration at the lowest frequencies in our model S0 of the eye that correspond to the same transversal cut as shown in Fig 1. Light and dark blue (red and yellow) shades indicated a motion towards (away from) the reader and normal to the drawn plane. *Left panels*: eigenfunctions with even parity in *l*: (*n* = 1, *l* = 2) vibrating at 318 Hz, (1, 4) at 648 Hz and (2, 2) at 909 Hz. *Right panels*: eigenfunctions with odd parity: (1, 3) at 492 Hz, (2, 1) at 1159 Hz and (1, 5) at 797 Hz.

In Fig 3 we show six different patterns of toroidal vibrational modes at the lowest frequencies in our simplified model of the eye that correspond to the same transversal cut as shown in Fig 1 (for a similar figure but considering spheroidal modes, see S2 Fig). The different patterns are identified by a set of two integer numbers *n* and *l* that denote the number of nodes the solution has in the radial and in the *θ*−angular direction, respectively. Each pair of values (*n*, *l*) has a unique characteristic frequency. The upper left panel of Fig 3 corresponds to matter rotating (counter-rotating) about the symmetry axis in the northern (southern) hemisphere (see S1 Fig for a three-dimensional representation of the mode (1, 2)). There is a number of normal mode frequencies falling in the range ∼ 100 Hz to ∼ 10 MHz (Table 1). Modes with frequencies of a few hundreds of Hz have periods of oscillation much shorter than other quasi-periodic variations of the eyeball volume triggered by phasic processes like respiration and pulse.

**Table 1:** Frequencies of selected normal modes of the simplified human eye.

T					S				
		*l*					*l*		
		1	2	3			1	2	3
*n*	1	734.46	318.71	492.48	*n*	1	2835.9	5706.5	8569.3
	2	1159.0	909.36	1076.1		2	491.41	947.85	1364.7
	3	1570.3	1339.9	1514.1		3	339.58	694.99	1130.0

*Left*: Table containing the frequencies (measured in Hertz) of selected toroidal modes (T) computed with our numerical code for an average human eyeball (model S0). The set of material parameters employed to obtain these values are *R* = 0.0125 m, *ρ* = 1000 kg m^−3^, *E* = 0.2985 MPa, and *σ* = 0.49. Toroidal modes with *n* = 0 are forbidden since they require driving external forces (assumed non existing in this model). *Right*: Same as the left table for spheroidal modes (S).

#### Application of the method to improved eyeball models

**Fig 4:**
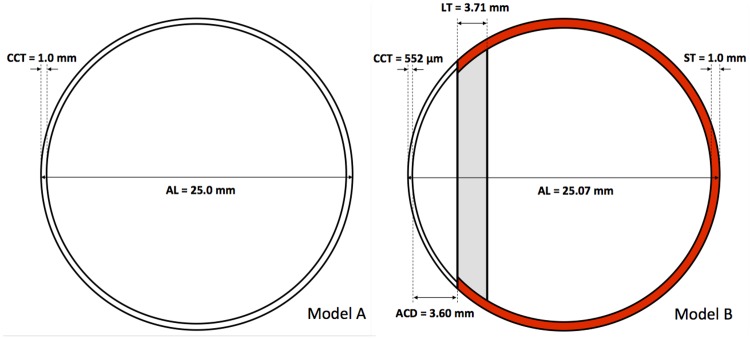
Improved eyeball models. Left: model A, where the spherical shell limiting the eyeball represents the cornea/sclera of the eye, holding a Young’s modulus much larger than the interior (*E*_s,c_ = 15 MPa, *E*_in_ = 0.2985 MPa). Right: model B, where we include a simplified model for the structure formed by the lens, lens capsule, suspensory ligaments and iris (gray shaded region). Also the cornea and the sclera (red colored) are differentiated.

Without abandoning the axial symmetry of our model, we have increased the degree of realism of our simplified eyeball by incorporating different viscoelastic properties to different constituents of the eye. In this section we consider two additional models in which the density of all the constituents is *ρ* = 1000 kg m^−3^, unless specified otherwise. In model A we incorporate an spherical shell with an outer radius *R* = 0.0125 m and a thickness of 1 mm (Fig 4, left). In model B, holding an outer radius *R* = 0.012035 m, we distinguish between the cornea and the sclera and we model the lens, the lens capsule, the ciliary body, the suspensory ligament and the iris altogether as a simple cylindrical region located at a distance *ACD* = 3.6 mm from the cornea (measured along the symmetry axis; see the gray-shaded region in Fig 4, right). The thickness of this region is *LT* = 3.71 mm, its Young’s modulus *E*_LT_ = 1 MPa, its Poisson ratio *σ*_LT_ = 0.47 and its density *ρ*LT = 1050 kg m^−3^. For the corneo-scleral layer of model A, we have chosen an average value for the Young’s modulus and Poisson ratio of *E*_s,c_ = 15 MPa and *σ*_s,c_ = 0.42, respectively. For model B, we keep the same Poisson ratio as in model A (*σ*_c_ = σ_s_ = 0.42), but modify the Young’s modulus and the thickness of the cornea (*CCT*), and of the sclera (*ST*). These values are *E*_c_ = 1 MPa and *CCT* = 0.552 mm for the cornea, and *E*_s_ = 45 MPa and *ST* = 1 mm for the sclera. The thickness values of the cornea and the location of lens with respect to the corneal center (equivalently, the width of the anterior chamber), its thickness and the axial length have been taken from recent *in vivo* measurements performed by our group [32]. The typical values of the Young’s modulus and Poisson ratio of the sclera and of the cornea have been obtained from Hugar & Ivanisevic [3]. The medium filling the interior of either models A or B is characterized by a Poisson ratio and Young’s modulus of *ν*_in_ = 0.49 and *E*_in_ = 0.2985 MPa, respectively.

The identification of the mode numbers (*n*, *l*) is an involved task in model B, where the spherical symmetry is lost. This is specially true for the spheroidal modes. Thus, we list in Table 2 the 10 lowest frequencies of each kind (toroidal or spheroidal) obtained with our numerical method, without identifying the vibrational numbers (*n*, *l*) they correspond to. We note, however, that the lowest frequencies usually correspond with the lowest allowed values of (*n*, *l*).

**Table 2:** Frequencies of selected normal modes of improved eyeball models.

model A		model B	
Toroidal	Spheroidal	Toroidal	Spheroidal
41	107	193	85
47	144	355	184
52	159	431	267
147	210	452	482
180	225	483	538
211	245	583	626
241	335	610	702
333	379	625	736

Table containing the lowest frequencies (measured in Hertz) of toroidal and spheroidal modes computed with our numerical code for the model A (columns 1 and 2) and for the model B (columns 3 and 4) of the human eyeball. The errors in the calculation of the vibrational frequencies for these two models are of the order of ∼ 10%–15% (a bit larger than those of model S0). The set of material parameters employed inside of the eyeball to obtain these values are *ρ* = 1000 kg m^−3^, *E* = 0.2985 MPa, and *σ* = 0.49, while the outer radius is *R* = 0.0125 m and *R* = 0.012035 m for the models A and B, respectively. For the properties of the surrounding layer see Sec. Application of the method to improved eyeball models.

We observe that the frequencies reported have become substantially smaller than those of the spherically symmetric model S0. In both improved models the frequency decrease is fundamentally triggered by the presence of a thin layer representative of the cornea/sclera. In model B, the presence of the lens with a reduced Poisson ratio and Young’s modulus places and “obstacle” in the inner resonant cavity added to the thicker sclera and cornea of this model. Both elements reduce effectively the inner cavity radius and, thereby, result in a frequency increase of the normal modes. Noteworthy, the values of the frequencies obtained with improved models are closer to other preexisting models in the literature [33], fact that we take as a hint of the robustness of our approach.

Toroidal modes have not been typically considered as relevant in the previous literature, but we they have eigenfrequencies in a similar range that spheroidal ones. However, toroidal modes begin at frequencies a factor ∼ 2 larger than those corresponding to spheroidal vibrations in the more realistic models A and B. This is in clear contrast with the situation described for model S0, where the toroidal eigenfrequencies are typically smaller than the spheroidal ones.

## Discussion

In the following, we discuss first the limitations of our current model (Sect. Model limitations). Next, we compare our model with some earlier attempts to reconstruct the mechanical properties of the eyeball from the vibrational properties of the cornea (Sect. Comparison to previous work). Then, we analyze the prospects to measure the vibrational modes of the eye with existing technologies that where devised for different purposes, but can be suitably adapted (Sect. Methods to measure the eigenfrequencies of the eye).

### Model limitations

The current notion is that the eyeball constituents are complex anisotropic composites, with nonlinear elastic and viscoelastic properties and highly heterogeneous [34]. However, a detailed 3-dimensional description and modeling of such a complex system is hardly possible nowadays. Thus, we are forced to reduce the complexity by assuming that the eyeball constituents possess linear, piece-wise isotropic properties in order to formulate a problem with a feasible solution in terms of material science.

A more accurate modelling of the eye structure than the one presented in the Sect. Materials and methods requires differentiating (at least) between the eye interior (including the lens and the aqueous humour) and its elastic boundary (the cornea and the sclera). In our model, this has been done assigning different elastic properties to different parts of the eye. Indeed, it is possible to assign different elastic properties on a point-by-point basis, to account for the heterogeneity of the various eye constituents. The results of more elaborated models have been presented in Sec. Application of the method to improved eyeball models. In spite of the added complexity, the degree of realism of the more sophisticated models is not optimal, but in this paper we have shown the path for easily improve them. Here, our goal is to outline that the analysis of the normal modes may provide useful mechanical information of the eyeball. If we could measure variations in the eyeball structure and if they could be attributed to normal modes, it would be possible to set an inversion problem [35] to obtain, for instance, the elastic moduli of the eye. The accuracy of the solutions obtained by the inversion problem depends on the number of properly identified eigenmodes and on the degree of realism in the model of the eyeball. As working hypothesis we assume that variations in the intraocular pressure (IOP) can be used as tracers of the eyeball volumetric changes induced by (spheroidal) normal modes of the eye. A lot of work has been done to connect the dynamics of the intraocular fluid by specifically modelling the aqueous humour as a hydrodynamic system where the inflow/outflow balance of such humour sets its physical properties, including the IOP [36]. The variations of the intraocular blood volume can be produced by many factors, the foremost being pulse, respiration, IOP fluctuations, and nervous mechanisms. The arteries of the eye are thick-walled and relatively inelastic; thus the influence of pulse pressure on intraocular pressure is heavily damped [37]. Contrarily, the venous system is thin-walled and easily collapsible and hence, its volume can sensitively change, though in a tiny amount compared to the full eyeball volume [38]. We also point out that other works have attempted to model only the vitreous humour as a viscoelastic fluid, considering the vitreous chamber as a sphere, and assuming only the effect of toroidal modes (see [33] and references therein). Different from these works, we also compute possible radial modes and present a general method that can be adapted to arbitrary geometries.

Our model needs to be ultimately calibrated with the acquisition of actual data of the eyeball. The ability to measure the changes in the eyeball shape resulting from spheroidal normal modes by mechanical means strongly relies on the maximum amplitude of the deformations induced. In practice, the amplitude of the modes will depend on the amplitude of the perturbations applied to the eye. As we will show in the next section, devices developed for the continuous monitoring of the IOP variations in glaucoma treatment [39] can be used to measure the temporal variations of the eyeball volume (and, thus, its normal modes). Since the inner eye constituents are nearly incompressible, the spherical elastic outer shell comprised of the sclera and the cornea must stretch to accommodate their respective volume changes. The volume of the eyeball may change as a result of the finite compressibility of intraocular tissue (iris, lens, ciliary body) or due to intraocular muscular contraction. However, these latter effects are secondary, and it is primarily the distension of the wall of the eyeball by its incompressible contents that governs the IOP [38].

### Comparison to previous work

We point out that there are some remarkable efforts along the direction of measuring the mechanical properties of different types of eyeballs (not exclusively human) employing the vibrational properties of the eyeball constituents. For instance, employing pulses of ultrasound radiation at frequencies between 100 and 200 Hz, Zhang et al. [19] have been able to measure the mechanical properties of *ex-vivo* bovine corneas. Zhang’s work and, more generally speaking, Lamb wave dispersion ultrasound vibrometry methods [40], stem from a concept similar to ours: a radiation source induces Lamb waves (propagating inside of the cornea) whose frequencies can be computed numerically from a suitable dispersion relation as an eigenvalue problem. Certainly, simplifying approximations need to be done in this kind of approaches, in as much as Lamb waves are solutions for infinitely thin layers, while the cornea and the sclera have finite thickness. Comparing our work to that of Zhang et al. [19] is not straightforward, since our model includes not only the cornea but also the (simplified) interior structure of the eyeball. As expected (due to the larger size of the resonance cavity in which we compute our models), the solutions to the dispersion relation for Lamb waves propagating in thin layers mimicking the cornea tend to possess smaller vibrational frequencies than those obtained in our model.

The finite element viscoelastic model of Kling et al. [21] was verified experimentally in flaps from bovine corneas and in porcine eyes employing sound excitation in the range 100–110 dB together with phase-sensitive optical coherence tomography in order to measure the frequency response function, expected to yield observable vibrations in the range 50–510 Hz. Simulations showed that corneal vibration in flaps is sensitive to geometrical as well as biomechanical parameters, whereas in whole globes it is primarily sensitive to corneal biomechanical parameters only. Employing an ultra-high-speed Scheimpflug camera and taylor-made image processing algorithms, Koprowski et al. [41] have recently confirmed the existence of corneal vibrational models with harmonic frequencies of 54.3 Hz, 131 Hz, 232 Hz, 369 Hz and so on up to 458 Hz (i.e., values compatible with the ones reported in [21]). In our language, these frequencies correspond to the normal frequencies of radial modes, since they result from the measurements of corneal displacements with respect to their equilibrium position. Our models A and B display several frequencies in a range fully compatible with the measurements quoted by [41], considering that both their data and our eigenfrequencies may be affected by errors ∼ 10%–20%.

Akca et al. [42] proposed a finite element model that included an inner incompressible fluid bounded by a viscoelastic material with a shape very similar to that of bovine eyeballs. These authors identified three eigenfrequencies of radial vibrations of the cornea: 86 Hz (fundamental mode with (*n*, *l*) = (0, 1)), and 200 Hz and 310 Hz for the harmonic modes (0, 2) and (0, 3), respectively. The frequency range of obtained in [42] is slightly below the one presented in our more elaborated human eyeball models. The main reasons for the discrepancy where the larger radii of the bovine eyeballs (*R* = 0.0175 m) as well as the smaller values for assumed the corneal and scleral Young’s moduli (*E*_c_ = 37 kPa and, *E*_s_ = 79 kPa, respectively). We have computed also a model with parameters as defined in Acka et al. [42]. Among lowest frequencies of the radial modes we find 67 ± 10 Hz, 186 ± 24 Hz, 307 ± 45 Hz. Therefore, our results are also compatible with those of paper [42] within the error intervals at hand.

In analogy to our refined finite differences models A and B, there are a few papers which consider a finite element eyeball model coupled to an internal fluid [43, 44]. The model of Salimi et al. [44] consists of an spherical shell structure coupled to a an inviscid pressurized fluid devoid of any mean flow, which is filling it. Salimi S. et al. [44] showed that the frequency response of their fluid-solid coupled system exhibits an increase in the normal frequencies in response to the internal pressure growth. A relevant conclusion of Salimi et al. work is that the interaction between the inner fluid and the surrounding shell becomes important in order to accurately predict the system dynamics. This is certainly the case also for us, since we observe that, as the model is sophisticated including different regions with different mechanical properties, the normal eyeball modes display noticeable changes.

### Methods to measure the eigenfrequencies of the eye

Devices currently employed for the continuous measurement of the IOP or suitable upgrades thereof can be used to measure the eyeball spheroidal normal modes. There are intraocular sensors that require surgical implantation, e.g., telemetric pressure transducer systems [45], which have an acquisition rate of ∼ 500 Hz, which may suffice for the purpose of measuring the lowest frequency spheroidal modes in a human eye. So far, the high-frequency IOP fluctuations have been attributed to measurement noise [45]. Yet, our results suggest the possibility that they are (partly) produced by the eyeball eigenfrequencies.

Among the least invasive devices to monitor the IOP soft contact lens sensors [46] (CLS) seem to be promising for the detection of normal spheroidal modes. The CLS measure at rates of 10 Hz. This acquisition rate is insufficient to detect the volume variations induced by spheroidal modes. Likely, faster measurement rates are technically plausible. However, such ability is possibly not employed because there was no reason to provide a finer coverage of the IOP variations so far. Should it be technically viable to improve the data acquisition rate in CLS based devices, they could be used for the purpose of measuring the eye’s normal mode eigenfrequencies. Yet another minimally invasive way of measuring spheroidal normal modes is the pulse Doppler technique, which has been used to track Lamb waves propagating in the cornea [19]. Recently, Akca et al. [42] have presented an experimental methodology to record the vibrations of the cornea, and to derive its mechanical properties. The stimuli source was a speaker (rather than an imposed displacement) with frequencies sweeping almost the same range as the current study.

The idea of measuring the elastic properties of the human eye taking advantage of its internal motions has been treated from different perspectives in the literature. The scattering pattern of attenuated laser sources on the retina has allowed measuring the motion of the vitreous humour [47]. The shear elastic modulus could be determined from these observations [47]. Walton et al. [48] employed ultrasound films of eyes undergoing impulsive rotations and tracked the speckles present in the vitreous humour. Hence, the same technique can be used to measure toroidal normal modes, which have not received the same attention so far. The topic has recently gained momentum since the knowledge of the mechanical properties of the vitreous humour is instrumental for finding materials that can be used as vitreous substitutes [1]. Bonfiglio et al. [1] find resonances between the forcing frequency of their device and the artificial vitreous. Remarkably, high frequency resonances may result in undesirably large values of the stress acting on the retina yielding retinal detachments in extreme cases. These resonances are similar to the toroidal eigenmodes we consider here. The frequencies of some of the resonances are above 100 Hz, in line with our results.

Toroidal normal modes of the eye are incompressible and, hence, do not leave a trace on the IOP. However, toroidal normal modes are potentially accessible by alternative devices. The IOLMaster 700 SSOB has shown an excellent performance [32, 49, 50]. Should they take measurements at high enough rate, SSOBs could be used to identify toroidal normal modes. As the later modes yield axial displacements about the eyeball axis, they may produce (tiny) variations of the light propagating in a moving medium [51]. Light rays propagating in a whirling fluid remain straight. The travel times of rays that propagate with or against the flow differ by a characteristic number. The light rays differ by a certain phase. Consequently, light waves that move with or against the medium will show a distinct interference pattern in analogy to the Aharonov-Bohm effect of electrically charged matter waves [52]. Perhaps it will be possible in the near future to employ this effect in optical biometers permitting the measurement of internal displacements of the inner constituents of the eye. The non-invasive character of swept-source optical biometers (SSOBs) is the utmost advantage over alternative techniques for biometric data acquisition [53]. We foresee that the technical capabilities of SSOBs can be improved to obtain high frequency data acquisition of the size of distinct eye structures. Then, they could be used to identify the displacements of the internal constituents of the eye and, therefore, to try to set an inversion problem to recover their normal mode toroidal eigenfrequencies.

Pulse and respiration are periodic phenomena. Therefore, they neither affect the mean IOP nor the eyeball average volume. The typical frequencies of pulse and respiration are below 2 Hz and, thereby, they yield quasi-periodic displacements of the vascular system which can be distinguished from the computed eyeball eigenfrequencies (typically above 100 Hz). Furthermore, in order to trigger the normal eyeball modes, it is optimal to employ perturbations having frequencies as close as possible to the eigenfrequencies. The perturbations induced by pulse and respiration may fall short for this purpose if the (non-linear) mode coupling is weak. Micro saccadic motions of the eye can potentially trigger normal modes, since they happen at frequencies of up to ∼ 60 Hz, typically last 20–200 ms and their rotational peak speeds can be as large as 1000 deg/sec [54]. Micro saccades follow the saccadic main sequence, suggesting a common generator for micro saccades and saccades [54]. Micro saccadic motions are triggered by oscillatory motions of suitable frequencies [55]. The lowest frequencies of the normal modes of our model are close to the observed micro saccadic frequencies, or even closer to measured tremors, which consist of very fast (∼ 90 Hz), extremely small oscillations (about the diameter of a foveal cone) superimposed on drifts [54]. Employing loudspeakers with sufficient power is also a very promising technique [42] to stimulate the radial normal modes of the human eyeball.

## Conclusion

We have presented a novel way of performing the analysis of the normal modes of an idealized human eye importing the analytical results developed in a number of areas of Physics, more precisely in the field of Gravitational Wave Physics. Developing a simplified, spherically symmetric eyeball model, for which there exist analytic solutions for the eigenfrequencies, we have shown that our finite difference scheme is properly calibrated. Without lifting the assumption of spherical symmetry, we have resized our baseline model to the typical dimensions of human eyes. Additional refinements have been added to the model like, e.g., an outer crust that mimics the corneo-scleral layer of the eyeball (model A). Finally, a more elaborated, axially symmetric model has been developed, where a number of physiological components of the eye are incorporated in an idealized way (model B). The frequencies obtained for the fundamental and harmonic eigenmodes display a range of variation ≲ 15%. This small scatter in the obtained frequencies, together with the comparison with previously existing work shall be considered as a hint of the robustness and reliability of our approach.

While spheroidal (compressible) normal modes are broadly employed to characterize the mechanical properties of different eyeball constituents (specially the cornea), we find that toroidal (incompressible) normal modes have not been sufficiently deemed in the ocular biomechanics, with the notable exception of the numerical rheological studies of viscous flows in spherical cavities (e.g., [33]), which are conducted for the development of vitreous substitutes [1]. One of our goals in this paper has been to highlight the fact that toroidal modes are measurable and encode a valuable information to estimate the mechanical properties of the eyeball. This information is complementary to that contained in spheroidal modes and has been recognized in diverse fields of Physics (notably in Astrophysics and Geophysics).

Beyond the mechanical characterization of the eyeball components, the normal vibrational modes of the eye could be involved in physiological processes like, e.g., the accommodation. Accommodation occurs through changes in the shape and thickness of the crystalline lens. The thickness and the curvature of the lens increase, causing an increase in the eye’s optical power. Since it is a muscle-induced activity, accommodation is a highly fluctuant and dynamic process. These fluctuations are related to the fluctuations in ocular aberrations, and occur with corresponding frequencies [56–58]. The microfluctuations of accommodation play an important role in the variability of the optical quality of the eye. There are two main components of the accommodation response: a low frequency component (< 0.5 Hz), which corresponds to the drift in the accommodation response, and a peak at higher frequency, in the 1–2 Hz band [56, 57]. The vibrational eyeball modes we have considered –having the lowest frequencies– seem to happen on timescales of a few milliseconds. The exact way in which the normal eyeball modes are correlated with the accommodation process is beyond the scope of this paper. However, we anticipate that to tackle such study one would need to improve our current model. Towards this direction we will conduct our future research.

## Supporting information

S1 Appendix (PDF)

S1 Fig Three-dimensional representation of the toroidal mode *n* = 1 and *l* = 2. The mode displayed corresponds to the upper left panel of Fig 3. The arrows indicate the direction of the motion about the symmetry axis of the system (showed with a black arrow).
(TIFF)

S2 Fig Spheroidal modes. The number of radial (angular) nodes is annotated by *n* (*l*). *Left panels*: The eyeball spheroidal mode (0,1), corresponding to a purely radial mode vibrating at 2836 Hz, in three different moments of its oscillatory vibrational pattern encompassing a half displacement period. We illustrate a typical vibrational period, from maximum expansion (top left) to maximum compression (bottom left) along the horizontal axis. On the central panel the displacements everywhere in the eyeball are null. The bottom and top panels correspond to times of maximum radial displacement in the horizontal direction. The arrows mark the direction of the displacements. In these left panels it is possible to observe the radial displacement of the boundaries with respect to the equilibrium state. The maximum displacement of the eyeball boundary is ∼ 0.15 mm for the mode (0, 1), but this value is fixed for illustration purposes, since the displacement corresponding to a given normal mode frequency is an eigenfunction of the Navier-Cauchy operator, thus it possesses an arbitrary normalization. The quantification of the maximum radial displacements must be done measuring experimentally the variations of the eyeball shape. *Right panels*: Snapshots of different vibrational, spheroidal modes when the displacements are maximal. From top to bottom, we display the modes (*n*, *l*) = (0, 2), (1, 1) and (1, 2) oscillating at frequencies 5707 Hz, 491 Hz and 948 Hz, respectively. Black circumferences mark the location of the eyeball boundary in the relaxed state.
(TIFF)
