# Supplementary material for: Estimation of the mechanical properties of the eye through the study of its vibrational modes
Source: PLoS One. 2017 Sep 18;12(9):e0183892. doi: 10.1371/journal.pone.0183892 (PMC5603173; doi:10.1371/journal.pone.0183892)
Supplement: S1 Appendix — (PDF) [file pone.0183892.s001.pdf]

Estimation of the Mechanical Properties of the Eye through the Study of its Vibrational Modes

M. Á. Aloy[1,*◐], J.E. Adsuara[1◐], P. Cerdá-Durán[1◐], M. Obergaulinger[1◐], J.J. Esteve-Taboada[2◐], T. Ferrer-Blasco[2◐], R. Montés-Micó[2◐]

1 Department of Astronomy and Astrophysics. University of Valencia. Spain.
2 Department of Optics and Optometry and Vision Sciences. University of Valencia. Spain.

◐These authors contributed equally to this work.
* miguel.a.aloy@uv.es

Supporting Information

S1 Appendix. **Analytic normal modes.** In linear elasticity, the equation of motion for an homogeneous isotropic elastic solid is given by the *Navier-Cauchy* equation [1], which can be written either in vector form

$$(\lambda + 2\mu)[\nabla(\nabla \cdot \boldsymbol{u})] - \mu[\nabla \times (\nabla \times \boldsymbol{u})] + \boldsymbol{F} = \rho\ddot{\boldsymbol{u}}, \tag{1}$$

or, component-wise, as:

$$\mu\nabla^2 u_i + (\lambda + \mu)\vartheta_{;i} + F_i = \rho\ddot{u}_i, \tag{2}$$

where u_i are the *displacements* with respect to an equilibrium position, $\vartheta := \nabla \cdot \boldsymbol{u}$ is the *dilatation*, double dotted quantities denote the second time derivatives (∂^2_{tt}) of such quantities, ∇^2 is the Laplacian operator, F_i denote the body forces, and μ and λ are the *Lamé constants*. The Lamé constants are related with the Young's modulus, E, and the Poisson ratio, σ, by the following expressions:

$$\sigma = \frac{\lambda}{2(\lambda + \mu)}, \qquad E = \frac{\mu(3\lambda + 2\mu)}{\lambda + \mu} \tag{3}$$

We start assuming oscillatory solutions of the form

$$u_i = u'_i \cos{(pt + \xi)}, \tag{4}$$

where p is the angular frequency of the perturbation, u'_i are functions independent of the time t and the constant ξ is independent of the coordinates x_i and only modifies the phase of the vibration. Plugging the test solutions Eq. (4) into Eq. (2), one obtains in the absence of body forces ($F_i = 0$):

$$\mu\Delta u_i + (\lambda + \mu)\vartheta_{;i} + \rho p^2 u_i = 0, \tag{5}$$

which can be rewritten in the form of an eigenvalue problem

$$-\frac{\mu}{\rho}\Delta u_i - \frac{\lambda + \mu}{\rho}\vartheta_{;i} = p^2 u_i. \tag{6}$$

where the allowed frequencies of vibration and their corresponding displacements (i.e., distortions of the underlying homogeneous and isotropic structure) correspond to the

eigenvalues and eigenvectors of the Navier-Cauchy equation for given boundary conditions (imposed at the eyeball surface; Fig. 1).

There are exact solutions of Eq. (6) for solid elastic bodies in which simple boundary conditions are imposed. One example is a sphere where suitable surface tractions inhibit the momentum through its surface (traction boundary conditions). Under the assumption of axisymmetry and expressing their results in terms of spherical solid harmonics ω_n, ϕ_{n+1} and χ_n, Rue [27] obtained, based on Love [28], analytic solutions for the shapes of these vibrations,

$$u_i = \sum_n \Big[-\frac{1}{h^2}\frac{\partial}{\partial x_i}\Big\{\omega_n \psi_n(hr)\Big\} + \psi_n(\kappa r)\Big(\epsilon_{ijk} x_j \frac{\partial}{\partial x_k}\chi_n + \frac{\partial}{\partial x_i}\phi_{n+1}\Big) - \frac{n+1}{n+2}\psi_{n+2}(\kappa r)\kappa^2 r^{2n+5}\frac{\partial}{\partial x_i}\frac{\phi_{n+1}}{r^{2n+3}}\Big] \quad (7)$$

as well as for the vibrational frequencies of spherical bodies. Here, ϵ_{ijk} is the *Levi-Civita symbol* and

$$\psi_n(x) := \left(\frac{1}{x}\frac{\partial}{\partial x}\right)^n \frac{\sin x}{x}, \quad (8)$$

In axially symmetric systems, the eigenmodes can be classified in two groups: toroidal and spheroidal. Toroidal modes only involve motion about the symmetry axis sketched in Fig. 1. Toroidal modes are incompressible since they do not change the volume of the eyeball. In this first class of vibrations ω_n and ϕ_n vanish ($\omega_n = \phi_n = 0$), and the frequency, from which we compute the eigenvalues of the system, is given by:

$$p_n = 0 \quad \text{with} \quad p_n := (n-1)\psi_n(\kappa a) + \kappa a \psi_n'(\kappa a) \quad (9)$$

Spheroidal modes, implying displacements of the eyeball material in both radial and/or angular directions, are compressible. In this second class of vibrations χ_n vanish ($\chi_n = 0$). The frequency equation is given by:

$$b_n c_n - a_n d_n = 0 \quad (10)$$

with:

$$a_n := \frac{1}{(2n+1)h^2}[\kappa^2 a^2 \psi_n(ha) + 2(n-1)\psi_{n-1}(ha)], \quad (11)$$

$$b_n := -\frac{1}{2n+1}\Big[\frac{\kappa^2}{h^2}\psi_n(ha) + \frac{2(n+2)}{ha}\psi_n'(ha)\Big], \quad (12)$$

$$c_n := \kappa^2 a^2 \psi_n(\kappa a) + 2(n-1)\psi_{n-1}(\kappa a), \quad (13)$$

$$d_n := \kappa^2 \frac{n}{n+1}\Big[\psi_n(\kappa a) + \frac{2(n+2)}{\kappa a}\psi_n'(\kappa a)\Big]. \quad (14)$$

S1 Fig. Three-dimensional representation of the toroidal mode $n = 1$ and $l = 2$. The mode displayed corresponds to the upper left panel of Fig. 3. The arrows indicate the direction of the motion about the symmetry axis of the system (showed with a black arrow).

S2 Fig. Spheroidal modes. The number of radial (angular) nodes is annotated by n (l). *Left panels*: The eyeball spheroidal mode $(0, 1)$, corresponding to a purely radial mode vibrating at 2836 Hz, in three different moments of its oscillatory vibrational pattern encompassing a half displacement period. We illustrate a typical vibrational period, from maximum expansion (top left) to maximum compression (bottom left) along the horizontal axis. On the central panel the displacements everywhere in the

eyeball are null. The bottom and top panels correspond to times of maximum radial displacement in the horizontal direction. The arrows mark the direction of the displacements. In these left panels it is possible to observe the radial displacement of the boundaries with respect to the equilibrium state. The maximum displacement of the eyeball boundary is $\sim 0.15\,\mathrm{mm}$ for the mode $(0, 1)$, but this value is fixed for illustration purposes, since the displacement corresponding to a given normal mode frequency is an eigenfunction of the Navier-Cauchy operator, thus it possesses an arbitrary normalization. The quantification of the maximum radial displacements must be done measuring experimentally the variations of the eyeball shape. *Right panels*: Snapshots of different vibrational, spheroidal modes when the displacements are maximal. From top to bottom, we display the modes $(n, l) = (0, 2)$, $(1, 1)$ and $(1, 2)$ oscillating at frequencies 5707 Hz, 491 Hz and 948 Hz, respectively. Black circumferences mark the location of the eyeball boundary in the relaxed state.
