# Supplementary figures and images for: Estimation of the mechanical properties of the eye through the study of its vibrational modes
Source: PLoS One. 2017 Sep 18;12(9):e0183892. doi: 10.1371/journal.pone.0183892 (PMC5603173; doi:10.1371/journal.pone.0183892)

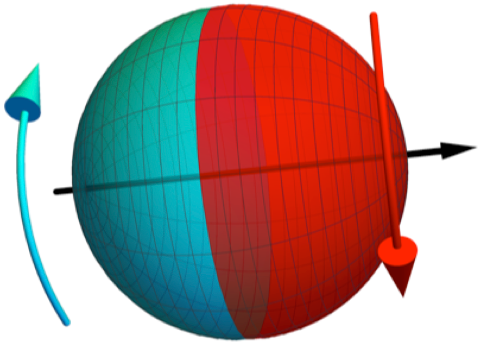

Supplement: S1 Fig — The mode displayed corresponds to the upper left panel of Fig 3. The arrows indicate the direction of the motion about the symmetry axis of the system (showed with a black arrow). (TIFF) [file pone.0183892.s002.tiff]

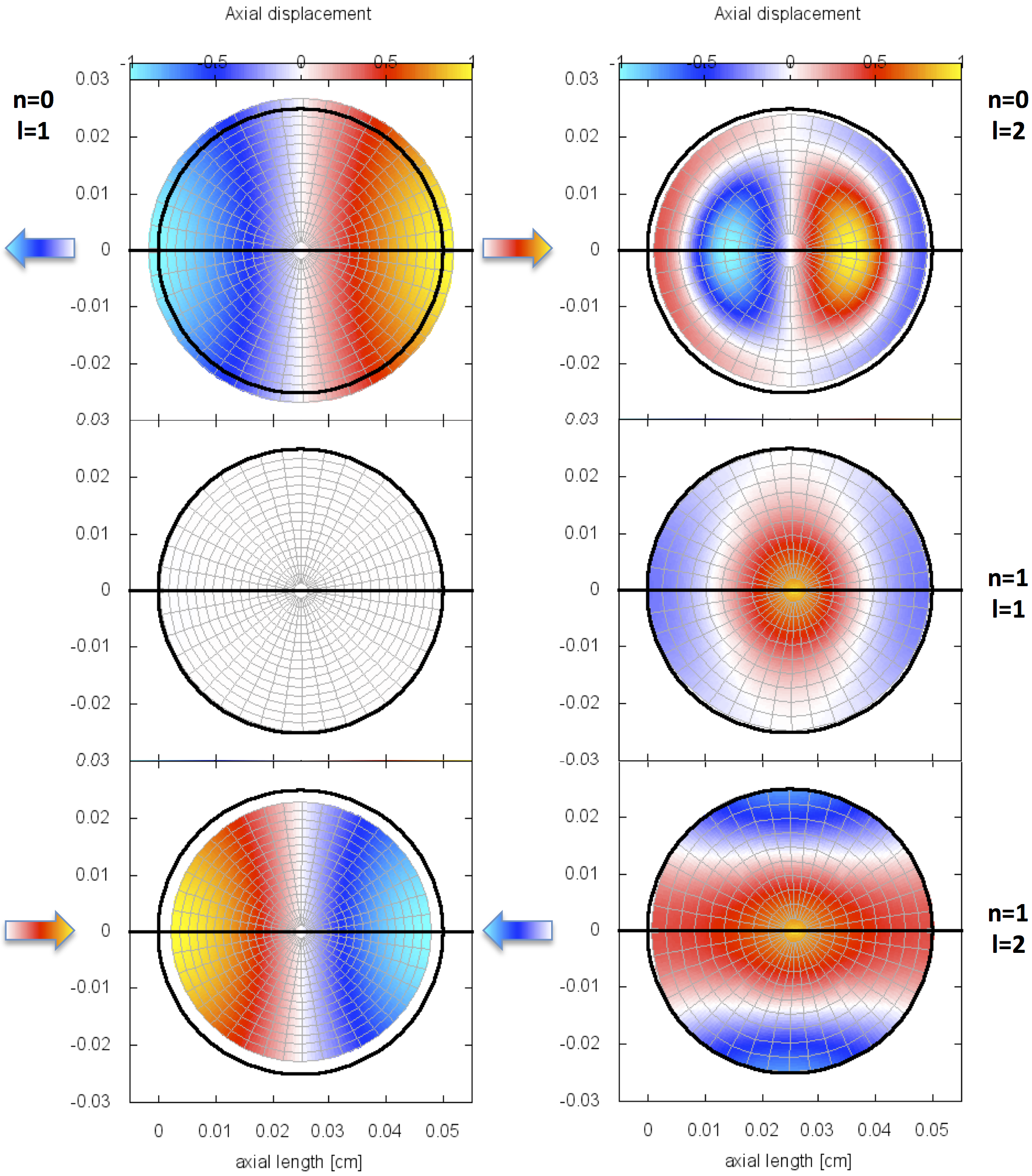

Supplement: S2 Fig — The number of radial (angular) nodes is annotated by n (l). Left panels: The eyeball spheroidal mode (0,1), corresponding to a purely radial mode vibrating at 2836 Hz, in three different moments of its oscillatory vibrational pattern encompassing a half displacement period. We illustrate a typical vibrational period, from maximum expansion (top left) to maximum compression (bottom left) along the horizontal axis. On the central panel the displacements everywhere in the eyeball are null. The bottom and top panels correspond to times of maximum radial displacement in the horizontal direction. The arrows mark the direction of the displacements. In these left panels it is possible to observe the radial displacement of the boundaries with respect to the equilibrium state. The maximum displacement of the eyeball boundary is ∼ 0.15 mm for the mode (0, 1), but this value is fixed for illustration purposes, since the displacement corresponding to a given normal mode frequency is an eigenfunction of the Navier-Cauchy operator, thus it possesses an arbitrary normalization. The quantification of the maximum radial displacements must be done measuring experimentally the variations of the eyeball shape. Right panels: Snapshots of different vibrational, spheroidal modes when the displacements are maximal. From top to bottom, we display the modes (n, l) = (0, 2), (1, 1) and (1, 2) oscillating at frequencies 5707 Hz, 491 Hz and 948 Hz, respectively. Black circumferences mark the location of the eyeball boundary in the relaxed state. (TIFF) [file pone.0183892.s003.tiff]
